# Avaliação da Senescência de Células Sanguíneas Mononucleares Periféricas e na Disfunção Endotelial entre Adultos com Alto Risco Cardiovascular

**DOI:** 10.36660/abc.20190409

**Published:** 2021-01-27

**Authors:** Vijay Raj, Soniya Charles, Luxitaa Goenka, Thilagavathi Ramamoorthy, C Marimuthu, C Emmanuel, Kanchana Mala, Subramaniyan Kumarasamy, Melvin George

**Affiliations:** 1 SRM Medical College Hospital Research Centre KancheepuramTamil Nadu Índia SRM Medical College Hospital and Research Centre - Medical Research, Kancheepuram, Tamil Nadu - Índia; 2 SRM Institute of Science and Technology KattankulathurTamil Nadu Índia SRM Institute of Science and Technology – Biotechnology, Kattankulathur, Tamil Nadu - Índia; 3 SRM Medical College Hospital Research Centre KancheepuramTamil Nadu Índia SRM Medical College Hospital and Research Centre - Clinical Pharmacology,Kancheepuram, Tamil Nadu - Índia; 4 SRM Institute of Science and Technology School of Public Health KattankulathurTamil Nadu Índia SRM Institute of Science and Technology - School of Public Health, Kattankulathur, Tamil Nadu - Índia; 5 Gleneagles Global Health City Chennai ChennaiTamil Nadu Índia Gleneagles Global Health City Chennai, Chennai, Tamil Nadu - Índia; 6 SRM Medical College Hospital Research Centre KancheepuramTamil Nadu Índia SRM Medical College Hospital and Research Centre - General Medicine,Kancheepuram, Tamil Nadu - Índia

**Keywords:** Doenças Cardiovasculares, Envelhecimento Celula, Endotélio, Biomarcadores, Pontuação de Propensão, Fatores de Risco

## Abstract

**Fundamento:**

Doenças cardiovasculares (DCV) são uma das principais causas de mortalidade e morbidade em todo o mundo. O envelhecimento biológico tem sido associado à ocorrência de resultados cardiovasculares. Entretanto, o mecanismo subjacente desse processo ainda é desconhecido.

**Objetivos:**

Buscamos avaliar se a senescência das células sanguíneas mononucleares periféricas (CSMP) e biomarcadores endoteliais poderiam influenciar o risco cardiovascular (CV) e ser marcadores adequados para a detecção precoce de doenças cardiovasculares em adultos.

**Métodos:**

Neste estudo transversal, pacientes livres de DCV foram classificados como baixo (n=32) e alto (n=28) escore de risco intracardaco (IHR) A senescência das CSMP foi avaliada estimando-se a atividade de telomerase (AT) e detectando-se a presença de células senescentes e disfunção endotelial, estimando-se a concentração de nitrito e nitrato e a capacidade antioxidante total (CAT). A análise estatística foi realizada com o software SPSS, versão 16.0 (SPSS Inc., Chicago, IL). Todos os p-valores <0,05 foram considerados estatisticamente significativos.

**Resultados:**

A senescência de CSMP de 0,95 [p-valor = 0,0001; 95% IC (0,874-1,026)] foi um indicador significativo de pacientes com escore de IHR mais alto, com um valor de corte de 21,65, com sensibilidade e especificidade de 92% e 88% respectivamente. Identificou-se que a senescência de CSMP, nitrito e nitrato, e AT eram independentemente associadas a um escore de IHR alto.

**Conclusão:**

Os status de nitrito e nitrato e AT, e a senescência de CSMP são medidas adequadas para prever o alto risco cardiovascular em adultos com risco CV. Entretanto devem ser realizados estudos de acompanhamento de longo prazo para confirmar esses achados. (Arq Bras Cardiol. 2021; 116(1):37-47)

## Introdução

Doenças cardiovasculares (DCV), tais como aterosclerose e infarto do miocárdio (IM) associado, continuam sendo uma das mais conhecidas e principais causas de mortalidade e morbidade em todo o mundo, inclusive na Índia. Além disso, os custos sociais e econômicos envolvidos no tratamento de DCV são altos. Estima-se que mais de 75% das mortes por causas cardiovasculares (CV) ocorrem em países com renda baixa e média.^1^ O envelhecimento cronológico é considerado um dos mais fortes indicadores da ocorrência de doenças CV e cerebrovasculares tais como IM, insuficiência cardíaca, aterosclerose e acidente vascular. Entretanto, o envelhecimento biológico pode ser considerado superior ao envelhecimento cronológico pela estratificação de risco de DCV.^2^ O processo de envelhecimento biológico refere-se particularmente ao acúmulo de danos endoteliais que ocorrem devido a vários mecanismos mecânicos, hemodinâmicos e imunológicos, e é determinado por fatores sociais e ambientais. Acredita-se que a senescência vascular (%) (SV), um tipo de envelhecimento biológico do sistema vascular tenha a relevância prognóstica e terapêutica na aterosclerose. O envelhecimento biológico tem sido associado à ocorrência de resultados CV adversos. Entretanto, o mecanismo subjacente desse processo ainda é desconhecido.^3^ Além disso, o envelhecimento arterial é o principal reflexo do envelhecimento biológico.^4 , 5^ A ausência de atividade de telomerase (AT) leva ao encurtamento dos telômeros, que é um determinante importante do envelhecimento biológico, levando a várias doenças vasculares. O termo disfunção endotelial se refere a várias condições patológicas que incluem a alteração das propriedades anticoagulantes e anti-inflamatórias do endotélio, a desregulação da modelagem vascular, e a deterioração da regulação do crescimento vascular. A disfunção endotelial leva a produção ou disponibilidade atenuada de óxido nítrico (NO) e causa a upregulation do stress oxidativo pelo aumento da produção de espécies reativas do oxigênio (ERO).^6^ A senescência celular demonstrou ser equivalente à senescência epitelial, e, portanto, à senescência vascular.^7^ 0 Na prática clínica atual, o risco de DCV é estimado e quantificado com base em fatores de risco convencionais, tais como idade, diabetes, hipertensão, tabagismo, hipercolesterolemia e histórico familiar de DCV. Entretanto, indivíduos portadores de DCV podem ter apenas um ou nenhum dos fatores de risco tradicionais, e há a possibilidade de que esses fatores de risco possam não explicar totalmente o avanço da doença. Portanto, a avaliação de outros fatores de risco não tradicionais e incomuns pode ajudar clínicos a prever o risco futuro de DCV.^8^ Levantamos a hipótese de que a senescência das células sanguíneas mononucleares periféricas (CSMP) e biomarcadores endoteliais poderiam influenciar o risco CV e poderiam ser marcadores adequados para a detecção precoce de doenças cardiovasculares em adultos com alto risco CV.

## Materiais e Métodos

### Desenho e Configuração do Estudo

**Figura 1:**
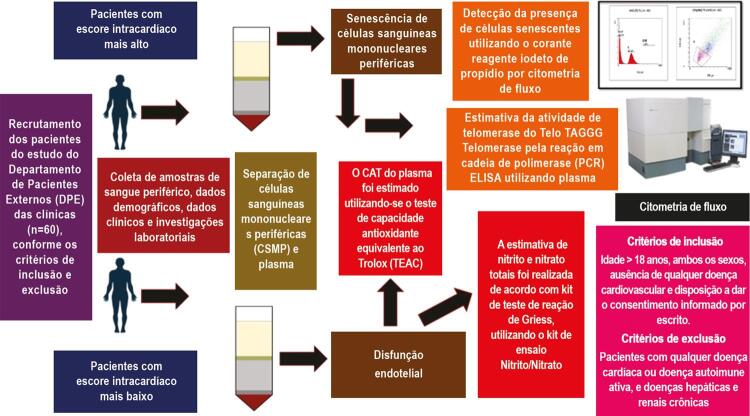
– Pacientes com escore de risco intracardíaco mais alto.

O protocolo do estudo foi aprovado pelo Comitê de Ética Institucional (973/IEC/2016). Todos os procedimentos do estudo foram seguidos de acordo com a Declaração de Helsinki. Todos os participantes deste estudo transversal passaram por triagem e foram recrutados no período entre janeiro de 2017 e dezembro de 2017, do departamento de pacientes externos (DPE) em Medicina Geral e das clínicas. A Figura 1 apresenta o delineamento do estudo de pesquisa.

### Sujeitos do Estudo

Incluímos todos os adultos acima de 18 anos de idade, de ambos os sexos, que compareceram ao DPE de Medicina Geral e clínicas sem quaisquer doenças cardíacas. Os pacientes com risco cardiovascular mais alto e mais baixo foram incluídos. Os pacientes foram classificados com base em seu escore de risco intracardíaco (IHR). O escore IHR foi calculado com base na presença ou ausência de fatores de risco CV conhecidos. Excluímos pacientes com quaisquer doenças cardíacas ou doenças imunes ativas e doenças crônicas do fígado e do rim.

### Escore de Risco Intracardíaco

Depois de obter o consentimento informado, os participantes do estudo passaram por triagem de acordo com os critérios de inclusão/exclusão e o escore de IHR foi medido. O escore de IHR foi calculado utilizando-se a versão que não incluía dados sobre níveis de colesterol. O escore de IHR, composto de informação sobre histórico médico e dados sobre idade, sexo, status em relação a diabetes, hipertensão, tabagismo, histórico familiar de doença cardíaca, razão cintura-quadril, fatores psicossociais, dieta e atividade física. Os escores de IHR variaram entre 0 e 48, em que escores mais altos ou mais baixos indicam escores de IHR mais altos e mais baixos, respectivamente. Um escore de IHR alto foi definido em > 16 unidades.^9^

### Coleta de Amostras

Foram retirados 3 ml de sangue da veia antecubital no antebraço em tubos vacutainers com heparina e ácido etilenodiamino tetra-acético (EDTA) separadamente. A amostra de sangue obtida nos tubos vacutainers com EDTA foi submetida e centrifugação a 2500 revoluções por minuto (rpm) por 10 minutos e o plasma isolado foi armazenado a -80°C. A amostra de sangue coletada nos tubos vacutainers com heparina foram processadas para separar a senescência de células sanguíneas mononucleares periféricas (CSMP), utilizando o reagente Ficoll-Histopaque. As CSMP isoladas foram fixadas com álcool 70% e foram armazenadas a 4°C para análises posteriores.^10^ A disfunção endotelial foi avaliada estimando-se a concentração de nitrito e nitrato e o status antioxidante.^11^

### Quantificação de Nitrito e Nitrato Totais

A estimativa de nitrito e nitrato totais foi realizada de acordo com kit de teste de reação de Griess, utilizando o kit de ensaio Nitrito/Nitrato (Sigma-Aldrich-Número no catálogo 23479, St. Louis, EUA), de forma a avaliar indiretamente a biodisponibilidade de óxido nítrico (NO). Filtros centrífugos com peso molecular de corte de 3.000 KDa foram utilizados para filtrar as amostras de plasma (300μl cada). A análise das amostras de plasma passante foi realizada utilizando-se microplacas para titulação de 96 poços, e a absorção foi medida a 540 nm em relação a padrões de referência.

### Estimativa da Atividade de Telomerase

A AT do plasma foi estimada usando-se a reação o TeloTAGGG Telomerase PCR ELISA (Ensaio imunoenzimático fotométrico para detecção da atividade de telomerase, utilizando o Protocolo de amplificação da repetição de telomerase (TRAP, do inglês *Telomerase Repeat Amplification Protocol* ), Roche Diagnostics GmbH, Roche Applied Science-Número do catálogo 11854666910, Mannheim, Alemanha]. O ensaio foi realizado de acordo com as instruções do fabricante.

### Estimativa da Capacidade Antioxidante Total (CAT)

O CAT do plasma foi estimado utilizando-se o teste de capacidade antioxidante equivalente ao Trolox (TEAC, do inglês *Trolox equivalent antioxidant capacity* ). A análise foi realizada de acordo com as instruções do fabricante fornecidas no kit de ensaio antioxidante disponível comercialmente (Sigma-Aldrich-Número do catálogo CS0790, St. Louis, EUA). Esse ensaio foi baseado na capacidade de determinar a presença de antioxidantes de peso molecular baixo no plasma inibe a produção de ABTS+ produzido pela oxidação de ABTS [2,2’- azino-bis(3-etilbenzotiazolina-6-ácido sulfônico)]. O CAT foi expresso na forma de equivalentes do Trolox (mM).

### Citometria de Fluxo (FACS)

As CSMP foram isoladas do sangue total utilizando o reagente Ficoll-Histopaque. Depois de isoladas, as CSMP foram fixadas com álcool 70% e foram armazenadas a 4°C durante a noite.^10^ As células isoladas foram, então, incubadas por 10 minutos com Rnase A (1mg/ml) por 10 minutos em temperatura ambiente. A senescência das CSMP (%) foram detectadas usando o reagente corante iodeto de propídio por citometria de fluxo (FC 500 Beckmann Coulter).

### Análise Estatística

A análise estatística do estudo foi realizada utilizando-se o software SPSS 16.0 (SPSS Inc., Chicago, IL, EUA), e p<0,05 foi considerado estatisticamente significativo. A normalidade dos dados para variáveis contínuas foi verificada, utilizando-se gráficos Q-Q. As variáveis contínuas foram resumidas como média ± desvio padrão (DP) e os dados categóricos foram expressos em frequência (porcentagens). Diferenças nas variáveis categóricas entre os grupos foram avaliadas com o teste qui-quadrado. Testes paramétricos foram usados com base na distribuição de dados. As diferenças em variáveis contínuas entre os grupos foram analisadas utilizando o teste t de amostras independentes. A correlação de Pearson foi realizada para identificar quaisquer associações entre as diferentes variáveis. Traçamos a curva de característica de operação do receptor (ROC) para identificar o corte para todos os ensaios de laboratório para prever o escore de IHR alto. Um escore de IHR alto foi definido em > 16 unidades.^9^ Todas as premissas necessárias pra realizar a análise de regressão linear foram cumpridas. Modelos de regressão múltipla foram plotados para determinar se as variáveis independentes senescência de CSMP, nitrito e nitrato, CAT e AT poderiam prever o escore de IHR.

## Resultados

### Características de Linha de Base dos Pacientes do Estudo

As características de linha de base dos pacientes do estudo foram ilustradas ( Tabela 1 ). Os pacientes do estudo (n=60) foram classificados em dois grupos de pacientes com escore de IHR mais baixo (n=32) e mais alto (n=28). Os pacientes com escore de IHR ≥16 foram classificados como pacientes com escore de IHR mais alto, e os com IHR < 16 foram classificados como de escore de IHR mais baixo. A média de idade dos pacientes do estudo com escores de IHR mais baixo e mais alto foi de 38,09±15,82 e 43,57±11,55 anos, respectivamente. Não houve diferença significativa quanto ao gênero dos grupos do estudo. Os escores médios de IHR entre os pacientes com escores de IHR mais baixo e mais alto foram 8,5±4,27 unidades e 20,46±2,19 unidades, respectivamente. Como esperado, a presença de fatores de risco de CV tais como diabetes e hipertensão foi maior entre pacientes com escore de IHR mais alto do que entre os com escore de IHR mais baixo.

**Tabela 1:** – Dados demográficos e fatores de risco dos participantes do estudo

No. Sl	Características	Sujeitos com IHR mais baixo (n=32)	Sujeitos com IHR mais alto (n=28)	p valor
1.	Idade, anos	38,09±15,82	43,57±11,55	0,13
2.	Sexo masculino, n (%)	20 (62,5%)	14 (50%)	0,33
3.	Escore de risco intracardíaco (IHR)	8,5±4,27	20,46±2,19	0,0001
4.	Tabagismo, n (%)	2 (6,2%)	5 (17,9%)	0,16
5.	Diabetes, n (%)	1 (3,1)	22 (78,6%)	0,0001
6.	Hipertensão, n (%)	32 (100%)	7 (25%)	0,003
7.	Histórico familiar de doença cardíaca, n (%)	2 (6,2%)	4 (14,3%)	0,30
8.	Sedentarismo, n (%)	8 (25%)	9 (32,1%)	0,54

*Os dados foram expressos como média ± desvio padrão e frequência (porcentagem). Os dados estatísticos usados para a comparação de variáveis contínuas foram t teste com amostras independentes, e, para as variáveis contínuas, o teste qui-quadrado. Um p-valor menor que 0,05 foi considerado estatisticamente significativo.*

### Senescência de Células Sanguíneas Mononucleares Periféricas

**Figura 2:**
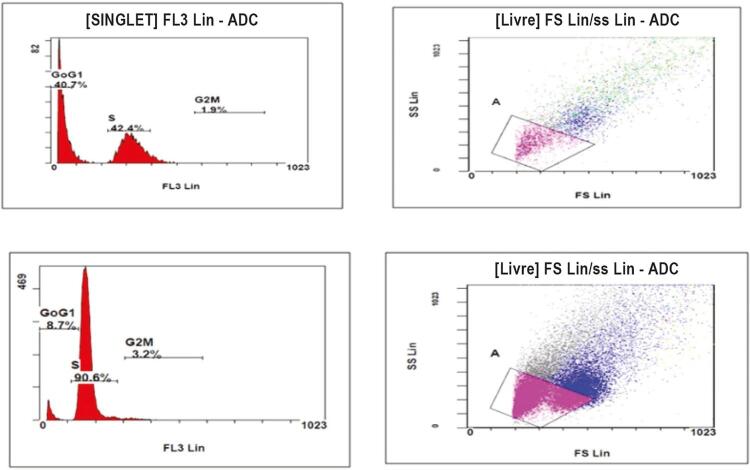
– Identificação e quantificação de células senescentes utilizando iodeto de propídio.

**Figura 3:**
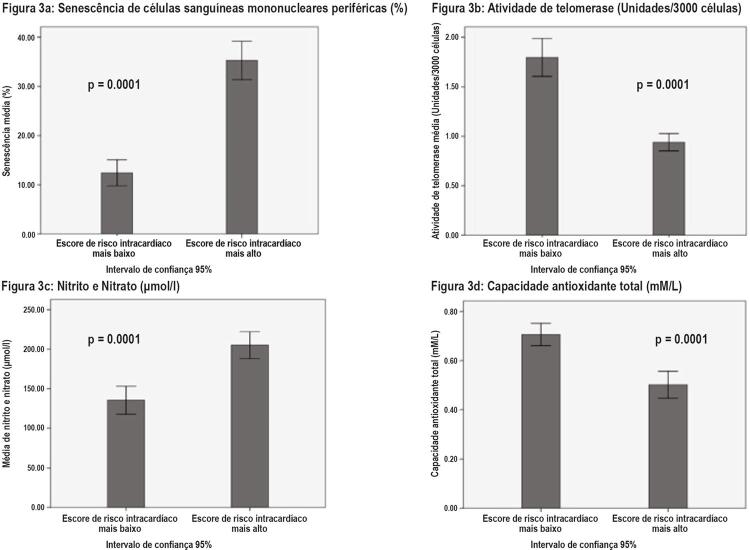
– Comparação da senescência de células mononucleares do sangue periférico, atividade telomerase, nitrito / nitrato e capacidade antioxidante total entre pacientes com escores de risco intermediário baixo e alto. O teste estatístico utilizado para comparar variáveis contínuas foi o teste T com amostras independentes; um p-valor menor que 0,05 foi considerado estatisticamente significativo.

A senescência das CSMP foi avaliada nos pacientes do estudo estimando-se a AT e detectando-se a presença de células senescentes ( Figura 2 ). A senescência média de CSMP (%), a porcentagem de células senescentes foi significativamente mais baixa entre os pacientes com escore de IHR mais baixo (12,41 ± 7,40) do que em pacientes do escore de IHR mais alto (35,26 ± 10,02) [p=0,0001] (( Figura 3a ). A média de AT (unidades/3000 células) foi significativamente mais alta entre pacientes com escore de IHR mais baixo do que com mais alto [(1,80±0,53 unidades/3000 células) versus (0,94±0,23 unidades/3000 células) [p=0,0001] ( Figura 3b ). A presença de fatores de risco cardíaco, tais como diabetes, hipertensão e tabagismo, influenciou os níveis de senescência de CSMP e AT ( Tabela 2 ).

**Tabela 2:** – Quantificação da senescência de células sanguíneas mononucleares periféricas e na disfunção endotelial baseada na presença e ausência de fatore de risco

	Senescência de CSMP		Nitrito e nitrato		Atividade de telomerase		CAT	
Fatores de risco	Presença	Ausência	Presença	Ausência	Presença	Ausência	Presença	Ausência
**Diabetes**	34,99±9,99*	15,67±11,44	204,22±42,39*	145,41±55,19	0,96±0,23*	1,67±0,60	0,52±0,08*	0,66±0,14
**Hipertensão**	40,37±10,68*	20,79±13,26	224,71±28,01*	160,45±56,84	0,81±0,18*	1,47±0,59	0,40±0,09*	0,64±0,12
**Tabagismo**	36,05+12,38*	21,36+13,83	204,14±56,40	163,17±56,97	0,88±0,23*	1,46±0,60	0,51±0,12*	0,65±0,13
**Doença cardíaca F/H/O**	31,40±16,81	22,15±13,96	191,17±36,08	165,37±59,58	1,04±0,43	1,44±0,60	0,48±0,15	0,63±0,13
**Sedentarismo**	28,86±15,33	20,79±13,50	172,47±53,74	166,16±60,06	1,24±0,52	1,45±0,62	0,57±0,14	0,63±0,13

*p<0.05; CSMP: Células sanguíneas mononucleares periféricas; CAT: Capacidade antioxidante total. Os dados foram expressos como média ± desvio padrão. Os dados estatísticos usados para a comparação das variáveis foram t teste com amostras independentes. Um p-valor menor que 0,05 foi considerado estatisticamente significativo.

### Disfunção Endotelial

A concentração de nitrito e nitrato foi ligeiramente mais alta entre pacientes com escore de IHR mais alto em comparação com pacientes com escore de IHR mais baixo [205,14±43,60 µmole/l versus 135,41±48,95 µmole/l (p=0,0001)] ( Figura 3c ). A CAT foi significativamente mais alta entre pacientes com escore de IHR mais baixo do que com mais alto [(0,71±0,08 mM/L) versus (0,50±0,09 mM/L) (p=0,0001] ( Figura 3d ). Entretanto a CAT foi estimada para apenas 30 sujeitos. Uma tendência semelhante foi observada entre pacientes fumantes, diabéticos e hipertensos ( Tabela 2 ).

### A relação entre Senescência de CSMP e Disfunção Endotelial

**Figura 4:**
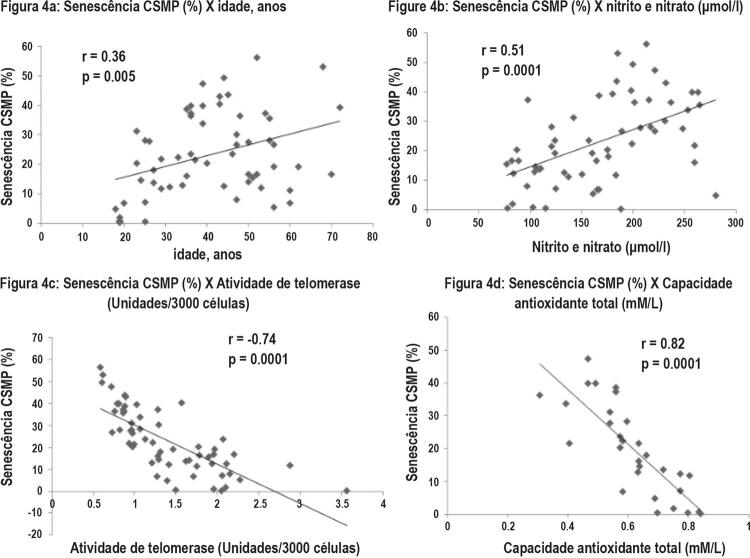
– Correlação da senescência de CSMP com idade, nitrito / nitrato, atividade telomerase e capacidade antioxidante total.

Observamos uma correlação positiva entre idade e senescência de CSMP (r=0,36, p=0,005), mas uma correlação negativa significativa foi observada entre idade e CAT (r=-0,60, p=0,0001). O escore de IHR demonstrou correlações positivas significativas com senescência de CSMP (r=0,75, p=0,0001) e nitrito e nitrato (r=0,56, p=0,0001), enquanto correlações negativas significativas foram observadas com AT (r=-0,83, p=0,0001) e CAT (r=-0,92, p=0,0001). Além disso, a senescência de CSMP também demonstrou correlações significativas com variáveis nitrito e nitrato, CAT e atividade de telomerase ( Figura 4 ).

### Análise de Curva ROC para Senescência de CSMP e Disfunção Endotelial:

**Figura 5:**
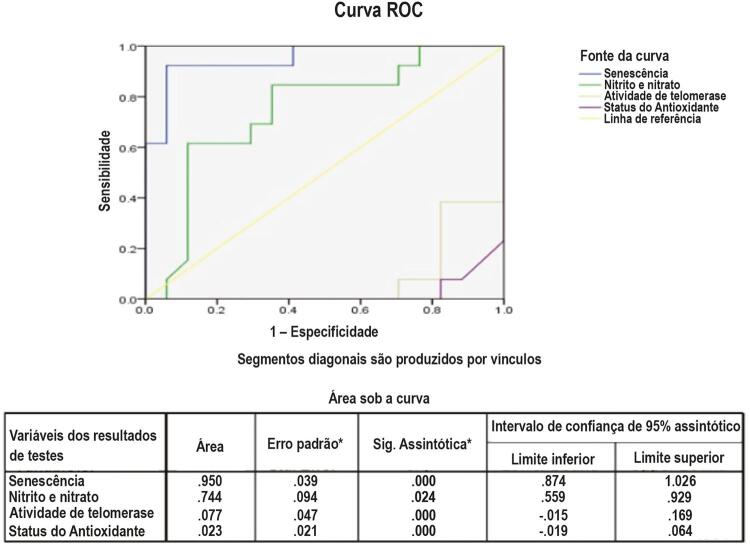
– Curvas de características operacionais do receptor para a previsão do High Interheart Risk Score.

A curva ROC foi traçada para verificar se senescência de CSMP, nitrito e nitrato, status de antioxidante e AT poderiam prever um escore de IHR alto entre os pacientes do estudo. A análise demonstrou que a senescência 0,95 [p-valor = 0,0001; 95% CI (0,874-1,026)] foi um indicador significativo de pacientes com escore de IHR mais alto, com um valor de corte de 21,65, com uma sensibilidade e especificidade de 92% e 88% respectivamente ( Figura 5 ).

### Modelos de Regressão Múltipla para Senescência de CSMP e Disfunção Endotelial

Modelos de regressão múltipla foram plotados para analisar os efeitos das variáveis independentes senescência de CSMP, nitrito e nitrato, e AT nas variáveis dependentes do escore de IHR ( Tabela 3 ). Observou-se que a senescência de CSMP, nitrito e nitrato, e AT eram independentemente associadas a um escore de IHR alto ( Tabela 3 ).

**Tabela 3:** – Modelos de regressão múltiplos para prever o escore de risco intracardíaco

		Coeficientes						
Modelo			Coeficientes não padronizados		Coeficientes padronizados	t	R quadrado ajustado	p valor
			B	Padrão Erro	Beta			
1	(Constante)		27,449	1,295		21,197	0,679	0,0001
	AtividadeTelomerase		-9,575	0,854	-0,827	-11,216		0,000
2	(Constante)		21,160	2,470		8,567	0,716	0,0001
	AtividadeTelomerase		-8,357	0,904	-0,722	-9,242		0,0001
	NitritoeNitrato		0,027	0,009	0,229	2,927		0,005
3	(Constante)		17,112	2,988		5,727	0,735	0,000
	AtividadeTelomerase		-6,608	1,169	-0,571	-5,652		0,000
	NitritoeNitrato		0,021	0,009	0,179	2,274		0,027
	Senescência		0,113	0,050	0,235	2,251		0,028

B: Coeficiente de regressão não padronizado; SEβ: Erro padrão de coeficiente; β: Coeficientes padronizados; p<0,05*

## Discussão

A relação entre senescência de CSMP e disfunção endotelial e a ocorrência de DCV foi descrita em estudos anteriores.^3 , 12^ Entretanto, as informações relacionadas à relação entre senescência de CSMP, disfunção endotelial, e DCV entre sujeitos sem qualquer DCV estabelecida continuam escassas. Este é o primeiro estudo clínico realizado na população do sul da Índia que estimou a senescência de CSMP e que determinou sua relação com alto risco de CV usando um escore de IHR. O principal achado de nosso estudo foi de que senescência de CSMP, nitrito e nitrato, e AT eram independentemente associados a um escore de IHR alto. A gravidade da senescência de CSMP foi maior entre pacientes com risco de CV mais alto se comparado a pacientes com risco de CV mais baixo. A senescência de CSMP foi estimada com base em AT e na porcentagem de células senescentes (%) entre os pacientes do estudo.

Telômeros e telomerase têm um papel significativo no desenvolvimento e na patogênese de DCV. Sabe-se que, a cada divisão celular, o comprimento dos telômeros diminui, enquanto a inflamação e o stress oxidativo, que são mecanismos importantes envolvidos no desenvolvimento e na patogênese de DCV aumenta o índice de encurtamento levando à senescência celular.^13 - 15^ Além disso, a presença de AT mais baixa e comprimento dos telômeros de leucócitos (CTL) foi vista em células endoteliais senescentes, células musculares lisas vasculares (CMLV), placa aterosclerótica, e estas também estão associadas com a instabilidade da placa que leva a DCV. A ausência de AT que mantém a integridade do telômero e o comprimento do telômero torna a célula senescente e causa apoptose.^16 - 18^ Nosso estudo revelou que a AT foi significativamente mais baixa em pacientes com escore de IHR mais alto do que mais baixo. Em contraste com nossos achados, um estudo anterior chamado desenvolvimento de risco da artéria coronária em jovens adultos (CARDIA), conduzido entre pacientes jovens com desenvolvimento de risco de artéria coronária com cálcio da artéria coronária (CAC), revelou que a AT desempenha um papel vital no desenvolvimento da aterosclerose. Os achados do estudo demonstraram que níveis mais altos de telomerase previram a prevalência mais alta de CAC entre homens de idade jovem a meia idade. Entretanto, os pacientes com telômeros mais curtos apresentaram uma associação positiva entre AT e CAC.^19^ Em um estudo transversal anterior, a associação entre carga de aterosclerose subclínica de um lado, e CTL médio e abundância de telômeros curtos (% CTL < 3 kb), de outro, foi estudada em 4.066 indivíduos de meia idade assintomáticos, sem a presença de qualquer DCV. O estudo demonstrou que o CTL médio e telômeros curtos não foram indicadores significativos e independentes de aterosclerose subclínica.^20^ Em um dos maiores estudos de observação e genéticos, conduzido em 290.022 indivíduos de Copenhagen, revelou-se que a presença de telômeros curtos foi associada a risco mais alto de doença cardíaca isquêmica.^21^A diferença nas constatações do estudo pode ser atribuída à heterogeneidade observada na população do estudo e do tamanho da amostra do estudo. Além disso, uma revisão sistemática recente e meta-análise de vinte e quatro estudos revelou uma associação inversa entre comprimento de telômeros de leucócitos e o risco de doença cardíaca coronária (DCC), independente de fatores de risco vascular convencionais.^3^ A revisão sistemática incluiu pacientes cardiovasculares, enquanto nosso estudo incluiu pacientes sem DCV. Portanto, pode-se sugerir que a medição de AT e CTL pode ser um marcador útil para prever o risco futuro de DCV. Atualmente, estão sendo realizadas investigações para medir se estatinas podem ser utilizadas como possíveis agentes terapêuticos para a ativação da telomerase e como geroprotetores eficientes.^22^

Recentemente, as células senescentes despertaram a atenção por seu potencial como alvo terapêutico para doenças relacionadas à idade, como as DCV. Estudos demonstraram que a senescência celular é equivalente à senescência epitelial, e, portanto, à senescência vascular. De aí em diante, o presente estudo mediu a porcentagem de células senescentes (%) que foi significativamente mais baixa com escore de IHR mais baixo em comparação com pacientes com escore de IHR mais alto. A análise transcricional de CMLV humanas demonstraram que houve supressão da proteína Gla da matriz (PGM), um inibidor de calcificação, nas CMLV senescentes. Além disso, houve a upregulation da transcrição que codifica a proteína óssea morfogenética 2 (BMP2), que promove a calcificação.^23^ Portanto, pode-se sugerir que as CMLV senescentes podem desempenhar um papel importante no desenvolvimento no endurecimento e no enrijecimento relacionados à idade, ao aumentar a calcificação. O enrijecimento e endurecimento das artérias levam ao desenvolvimento de pressão sanguínea alta, que é considerada um dos principais fatores de risco para a ocorrência de doença arterial coronariana, HF, acidente vascular, e IM.^24^ Outro estudo que foi realizado para comparar a expressão de PGM em células intersticiais da válvula aórtica (CIVA) normais versus doentes, que demonstrou que a expressão de PGM era significativamente reduzida nas CIVA doentes em relação às normais. Esses achados implicam que a ausência de um mecanismo de defesa contra a calcificação pode contribuir para a calcificação a válvula aórtica.^25^Portanto, a estimativa da porcentagem de células senescentes pode ser um marcador novo em potencial para indicar o desenvolvimento e o avanço de DCV. Estratégicas terapêuticas novas que envolvem a prevenção, a retirada e a substituição de células senescentes estão em fase inicial. É necessário ter melhor entendimento e realizar mais pesquisas para compreender a biologia para que esse conhecimento possa se traduzir em aplicações terapêuticas.

Neste estudo, a disfunção endotelial foi medida estimando-se a concentração de nitrito e nitrato e a CAT. A CAT foi ligeiramente mais baixa entre pacientes com escore de IHR mais alto em comparação com pacientes com escore de IHR mais baixo. Vários estudos epidemiológicos demonstraram que pessoas com maior consumo de vitaminas antioxidantes têm risco mais baixo de desenvolver IM e acidente vascular.^26 , 27^ Entretanto, uma revisão sistemática recente e meta-análise de ensaios controlados randômicos revelaram que a literatura atual não apresentava evidências para corroborar a hipótese do uso de vitaminas e antioxidantes para prevenir DCV.^28^Entretanto, uma revisão sistemática recente de estudos de observação demonstrou uma associação significativa entre níveis mais altos de capacidade de antioxidante total da dieta e fatores de risco de doenças cardiovasculares.^29^ Nosso estudo também demonstrou que as concentrações de nitrito e nitrato eram mais altas entre pacientes de alto risco em comparação com os de baixo risco. Em contraste, o estudo de Framingham demonstrou que uma concentração mais alta de nitrato no plasma foi associada a mortalidade global, mas não demonstrou estar associada à incidência de DCV.^30^ Isso pode se deter ao fato de que nitrito e nitrato presentes na dieta puderam ser metabolizados em NO e dessa forma promover a citoproteção e benefícios cardiovasculares.^31^ Os resultados em nosso estudo podem ser contrastantes, devido ao fato de que determinadas dietas, tais como vegetais, frutas e carnes processadas são fontes ricas em nitrito e nitrato.^32^ Portanto, há possibilidade de que pacientes de alto risco em nosso estudo estejam expostos a essas dietas. A resposta dependente do endotélio à vasodilatação é regulada pela liberação de NO sintetizado a partir de nitrato, nitrito e do aminoácido L-arginina da dieta, por meio de óxido nítrico-sintase endotelial (eNOS), que leva à produção de PGM cíclica intracelular. Entretanto, a disfunção endotelial leva ao desbalanço na produção de NO e ERO, levando à ocorrência de várias doenças relacionadas à idade, tais como as DCV. O acúmulo de ERO no plasma e intima arterial leva ao aumento da oxidação do lipoproteínas de baixa densidade (LDL); a captação desse LDL oxidado pelos macrófagos arteriais é um dos fatores importantes para a formação e avanço da placa aterosclerótica. Portanto, a presença de antioxidantes no plasma, partícula de LDL e parede da célula não podem inibir a oxidação do LDL, e podem proteger a vasorreatividade aumentando a liberação de NO endotelial e reduzindo a trombogenicidade.^12 , 33^Portanto, a determinação da CAT e da concentração de nitrito e nitrato pode acabar sendo um marcador potencial para previsão precoce de DCV no futuro.

### Limitações

A principal limitação deste estudo está relacionada ao tamanho limitado da amostra. Outra limitação é que este estudo não teve um acompanhamento prospectivo de longo prazo com a confirmação de eventos clínicos. Em vez disso, o risco foi calculado com base no escore de risco intracardaco. Além disso, as amostras de sangue foram coletadas em diferentes momentos, o que pode ter afetado os níveis de testes laboratoriais.

## Conclusões

Este estudo demonstrou que a senescência de CSMP, AT e nitrito e nitrato são medidas adequadas para prever risco cardiovascular alto em adultos com risco CV. Portanto, a medição dos marcadores acima pode ser utilizada como ferramenta adicional de avaliação de risco para prever o risco de doenças cardiovasculares entre adultos. Entretanto, estudos de acompanhamento prospectivos de longo prazo, com a avaliação de eventos clínicos, precisam ser realizados para confirmar esses achados.

## References

[1] . Appiah D, Capistrant BD. Cardiovascular Disease Risk Assessment in the United States and Low- and Middle-Income Countries Using Predicted Heart/Vascular Age. Sci Rep. 2017;7(1):16673. doi: 10.1038/s41598-017-16901-5. 10.1038/s41598-017-16901-5 PMC5709399 29192146

[2] . Niccoli T, Partridge L. Ageing as a risk factor for disease. Curr Biol. 2012;22(17):R741-52. doi: 10.1016/j.cub.2012.07.024. 10.1016/j.cub.2012.07.024 22975005

[3] . Haycock PC, Heydon EE, Kaptoge S, Butterworth AS, Thompson A, Willeit P. Leucocyte telomere length and risk of cardiovascular disease: systematic review and meta-analysis. BMJ. 2014;349: g4227. doi: 10.1136/bmj.g4227.. 10.1136/bmj.g4227 PMC4086028 25006006

[4] . Anderson R, Richardson GD, Passos JF. Mechanisms driving the ageing heart. Exp Gerontol. 2018; 109:5-15. doi: 10.1016/j.exger.2017.10.015. 10.1016/j.exger.2017.10.015 29054534

[5] . Iurciuc S, Cimpean AM, Mitu F, Heredea R, Iurciuc M. Vascular aging and subclinical atherosclerosis: why such a “never ending” and challenging story in cardiology? Clin Interv Aging. 2017; 12:1339-45. doi: 10.2147/CIA.S141265. eCollection 2017. 10.2147/CIA.S141265 PMC5574695 28883714

[6] . Hadi HAR, Carr CS, Al Suwaidi J. Endothelial dysfunction: cardiovascular risk factors, therapy, and outcome.Vasc Health Risk Manag. 2005;1(3):183-98. PMC1993955 17319104

[7] . Childs BG, Li H, van Deursen JM. Senescent cells: a therapeutic target for cardiovascular disease. J Clin Invest. 2018;128(4):1217-28. doi: 10.1172/JCI95146. 10.1172/JCI95146 PMC5873883 29608141

[8] . D’Agostino RB, Pencina MJ, Massaro JM, Coady S. Cardiovascular Disease Risk Assessment: Insights from Framingham. Glob Heart. 2013;8(1):11-23. 10.1016/j.gheart.2013.01.001 PMC3673738 23750335

[9] . InterHeart Risk Score-PHRI [home page on the Internet]. Medscape; 2018. [Cited 2018 May 15] Available from: https://rome.phri.ca/interheartriskscore

[10] . Dagur PK, McCoy JP. Collection, Storage, and Preparation of Human BloodCells.CurrProtocCytom. 2015;73(5):1-16 10.1002/0471142956.cy0501s73 PMC4524540 26132177

[11] . Del Ben M, Fabiani M, Loffredo L, Polimeni L, Carnevale R, Baratta F, et al. Oxidative stress mediated arterial dysfunction in patients with obstructive sleep apnoea and the effect of continuous positive airway pressure treatment. BMC Pulm Med. 2012; 12:36. doi: 10.1186/1471-2466-12-36. 10.1186/1471-2466-12-36 PMC3414800 22824065

[12] . Cai H, Harrison DG. Endothelial dysfunction in cardiovascular diseases: the role of oxidant stress. Circ Res 2000; 87:840-4. 10.1161/01.res.87.10.840 11073878

[13] . Wu J, Xia S, Kalionis B, Wan W, Sun T. The role of oxidative stress and inflammation in cardiovascular aging. Biomed Res Int. 2014; 2014:615312. doi: 10.1155/2014/615312. 10.1155/2014/615312 PMC4131065 25143940

[14] . Brouilette S, Singh RK, Thompson JR, Goodall AH, Samani NJ. White cell telomere length and risk of premature myocardial infarction. ArteriosclerThrombVasc Biol. 2003;23(5):842-6. 10.1161/01.ATV.0000067426.96344.32 12649083

[15] . O’Donnell CJ, Demissie S, Kimura M, Levy D, Gardner JP, White C, et al. Leukocyte telomere length and carotid artery intimal medial thickness: the Framingham Heart Study.ArteriosclerThrombVasc Biol. 2008;28(6):1165-71. doi: 10.1161/ATVBAHA.107.154849. 10.1161/ATVBAHA.107.154849 PMC3042248 18388332

[16] . Fuster JJ, Andrés V. Circ Res. 2006;99(11):1167-80. 10.1161/01.RES.0000251281.00845.18 17122447

[17] . Pepe S, Lakatta EG. Aging hearts and vessels: masters of adaptation and survival. Cardiovasc Res. 2005;66(2):190-3. 10.1016/j.cardiores.2005.03.004 15820187

[18] . Collins K. Mammalian telomeres and telomerase. CurrOpin Cell Biol. 2000;12(3):378-83. 10.1016/s0955-0674(00)00103-4 10801465

[19] . Kroenke CH, Pletcher MJ, Lin J, Blackburn E, Adler N, Matthews K et al. Telomerase, telomere length, and coronary artery calcium in black and white men in the CARDIA study. Atherosclerosis. 2012;220(2):506-12. doi: 10.1016/j.atherosclerosis.2011.10.041. 10.1016/j.atherosclerosis.2011.10.041 22178426

[20] . Fernández-Alvira JM, Fuster V, Dorado B, Soberón N, Flores I, Gallardo M et al. Short Telomere Load, Telomere Length, and Subclinical Atherosclerosis: The PESA Study. J Am Coll Cardiol. 2016;67(21):2467-76. doi: 10.1016/j.jacc.2016.03.530. 10.1016/j.jacc.2016.03.530 27230041

[21] . Scheller Madrid A, Rode L, Nordestgaard BG, Bojesen SE. Short Telomere Length and Ischemic Heart Disease: Observational and Genetic Studies in 290 022 Individuals. Clin Chem. 2016;62(8):1140-9. doi: 10.1373/clinchem.2016.258566. 10.1373/clinchem.2016.258566 27259814

[22] . Strazhesko ID, Tkacheva ON, Akasheva DU, Dudinskaya EN, Plokhova EV, Pykhtina VS et al. Atorvastatin Therapy Modulates Telomerase Activity in Patients Free of Atherosclerotic Cardiovascular Diseases. Front Pharmacol. 2016; 7:347. eCollection 2016. 10.3389/fphar.2016.00347 PMC5043056 27746733

[23] . Burton DGA, Giles PJ, Sheerin ANP, Smith SK, Lawton JJ, Ostler EL et al. Microarray analysis of senescent vascular smooth muscle cells: A link to atherosclerosis and vascular calcification. Exp Gerontol. 2009;44(10):659-65. doi: 10.1016/j.exger.2009.07.004. 10.1016/j.exger.2009.07.004 19631729

[24] . O’Rourke MF, Hashimoto J. Mechanical factors in arterial aging: a clinical perspective. J Am Coll Cardiol. 2007;50(1):1-13. 10.1016/j.jacc.2006.12.050 17601538

[25] . Venardos N, Bennett D, Weyant MJ, Reece TB, Meng X, Fullerton DA. Matrix Gla protein regulates calcification of the aortic valve. J Surg Res. 2015;199(1):1-6. doi: 10.1016/j.jss.2015.04.076. 10.1016/j.jss.2015.04.076 PMC4604002 25990696

[26] . Chen G-C, Lu D-B, Pang Z, Liu Q-F. Vitamin C intake, circulating vitamin C and risk of stroke: a meta-analysis of prospective studies. J Am Heart Assoc. 2013;2(6): e000329. doi: 10.1161/JAHA.113.000329. 10.1161/JAHA.113.000329 PMC3886767 24284213

[27] . Subhakumari K, Reshmy G, Sajitha Krishnan P. Evaluation of Antioxidant Status in Myocardial Infarction in Diabetic and Non-diabetic Subjects: A Comparative Study. Advanc Diabetes Metabol. 2015; 3:1–6.

[28] . Myung S-K, Ju W, Cho B, Oh SW, Park SM, Koo BK. Efficacy of vitamin and antioxidant supplements in prevention of cardiovascular disease: systematic review and meta-analysis of randomised controlled trials.BMJ. 2013;346: f10. doi: 10.1136/bmj. f10. 10.1136/bmj.f10 PMC3548618 23335472

[29] . Mozaffari H, Daneshzad E, Surkan PJ, Azadbakht L. Dietary Total Antioxidant Capacity and Cardiovascular Disease Risk Factors: A Systematic Review of Observational Studies. J Am Coll Nutr. 2018;37(6):533-45. doi: 10.1080/07315724.2018.1441079. 10.1080/07315724.2018.1441079 29714643

[30] . Maas R, Xanthakis V, Göen T, Müller J, Schwedhelm E, Böger RH et al. Plasma Nitrate and Incidence of Cardiovascular Disease and All-Cause Mortality in the Community: The Framingham Offspring Study. J Am Heart Assoc. 2017;6(11). pii: e006224. doi: 10.1161/JAHA.117.006224. 10.1161/JAHA.117.006224 PMC5721741 29151027

[31] . Tang Y, Jiang H, Bryan NS. Nitrite and nitrate: cardiovascular risk-benefit and metabolic effect. CurrOpinLipidol. 2011;22(1):11-5. doi: 10.1097/MOL.0b013e328341942c. 10.1097/MOL.0b013e328341942c 21102328

[32] . Hord NG, Tang Y, Bryan NS. Food sources of nitrates and nitrites: the physiologic context for potential health benefits. Am J Clin Nutr. 2009;90(1):1-10. doi: 10.3945/ajcn.2008.27131. 10.3945/ajcn.2008.27131 19439460

[33] . Tribble DL. AHA Science Advisory. Antioxidant consumption and risk of coronary heart disease: emphasison vitamin C, vitamin E, and beta-carotene: A statement for healthcare professionals from the American Heart Association. Circulation 1999;99(4):591-5. 10.1161/01.cir.99.4.591 9927409

