# Supplementary material for: Avaliação da Senescência de Células Sanguíneas Mononucleares Periféricas e na Disfunção Endotelial entre Adultos com Alto Risco Cardiovascular
Source: Arq Bras Cardiol. 2021 Jan 27;116(1):37–47. [Article in Portuguese] doi: 10.36660/abc.20190409 (PMC8159491; doi:10.36660/abc.20190409)
Supplement: Translation

# Translation: Assessment of Peripheral Blood Mononuclear Cells Senescence and Endothelial Dysfunction among Adults with High Cardiovascular Risk

**Authors:** Vijay Raj, Soniya Charles, Luxitaa Goenka, Thilagavathi Ramamoorthy, C Marimuthu, C Emmanuel, Kanchana Mala, Subramaniyan Kumarasamy, Melvin George

**Affiliations:** 1 India SRM Medical College Hospital and Research Centre - Medical Research, Kancheepuram, Tamil Nadu - India; 2 India SRM Institute of Science and Technology – Biotechnology, Kattankulathur, Tamil Nadu - India; 3 India SRM Medical College Hospital and Research Centre - Clinical Pharmacology, Kancheepuram, Tamil Nadu - India; 4 India SRM Institute of Science and Technology - School of Public Health, Kattankulathur, Tamil Nadu - India; 5 India Gleneagles Global Health City Chennai, Chennai, Tamil Nadu - India; 6 India SRM Medical College Hospital and Research Centre - General Medicine, Kancheepuram, Tamil Nadu - India

**Keywords:** Cardiovascular Diseases, Cell Aging, Endothelium, Biomarkers, Propensity Score, Risk Factors

## Abstract

**Background:**

Cardiovascular diseases (CVD) are one of the leading causes of mortality and morbidity worldwide. Biological aging has been associated with the occurrence of adverse cardiovascular outcomes; however, the underlying mechanism of this process remains unknown.

**Objectives:**

This study sought to evaluate if peripheral blood mononuclear cell (PBMC) senescence and endothelial biomarkers could influence cardiovascular (CV) risk and be suitable markers for the early detection of cardiovascular diseases in adults.

**Methods:**

In this cross-sectional study patients free of CVD were classified as lower (n=32) and higher Interheart Risk (IHR) scores (n=28). PBMC senescence was assessed by estimating the telomerase activity (TA) and detecting the presence of senescent cells and endothelial dysfunction by estimating the concentration of nitrite and nitrate and of total antioxidant capacity (TAC). Statistical analysis was performed with SPSS version 16.0 (SPSS Inc., Chicago, IL). All p-values <0.05 were considered statistically significant.

**Results:**

PBMC senescence 0.95 [p-value = 0.0001; 95% CI (0.874-1.026)] was a significant predictor of patients with higher IHR scores with a cut-off value of 21.65 with a sensitivity and specificity of 92% and 88% respectively. PBMC senescence, nitrite and nitrate and TA were found to be independently associated with high IHR scores.

**Conclusion:**

PBMC senescence, TA and nitrite, and nitrate status are suitable measures to predict high cardiovascular risk in adults with CV risk. Nevertheless, long-term follow-up studies are needed to confirm these findings. (Arq Bras Cardiol. 2021; 116(1):37-47)

## Introduction

Cardiovascular diseases (CVD) such as atherosclerosis and associated myocardial infarction (MI) are still one of the well-known and leading causes of mortality and morbidity worldwide, especially in India. Moreover, the social and economic costs incurred in the treatment of CVD are high. It has been estimated that more than 75% of the cardiovascular (CV) deaths occur in lower and middle-income countries.^1^Chronological aging is considered to be one of the strongest predictors for the occurrence of CV and cerebrovascular diseases, such as MI, heart failure (HF), atherosclerosis, and stroke; however, biological aging can be considered superior to chronological aging in the stratification of the CVD risk.^2^The process of biological aging particularly refers to the accumulation of endothelial damage, which occurs due to several mechanical, hemodynamic, and immunological mechanisms, and is determined by both social and environmental factors. Vascular Senescence (%) (VS), a kind of biological aging of the vascular system, is postulated to have prognostic and therapeutic relevance in atherosclerosis. Biological aging has been associated with the occurrence of adverse CV outcomes; however, the underlying mechanism of this process remains unknown.^3^Moreover, arterial aging is the primary reflection for biological aging.^4 , 5^The absence of telomerase activity (TA) leads to the shortening of telomeres, which is an important determinant of biological aging leading to several vascular diseases. The term endothelial dysfunction refers to a number of pathological conditions that include the altered anticoagulant and anti-inflammatory properties of the endothelium, dysregulation of vascular modelling and the impaired regulation of vascular growth. Endothelial dysfunction leads to attenuated production or the availability of nitric oxide (NO) and leads to the up-regulation of oxidative stress through the increased production of reactive oxygen species (ROS)^6^. Cell senescence has proven to be equivalent to endothelial senescence and thus vascular senescence.^7^In the current clinical practice, the risk of CVD is estimated and quantified on the basis of conventional risk factors such as age, diabetes, hypertension, smoking, hypercholesterolemia, and family history of CVD.^8^Nevertheless, individuals with CVD might have only one, or none of the traditional risk factors and there is a possibility that these risk factors might not fully account for the disease progression. Therefore, the evaluation of other non-traditional and uncommon risk factors might aid clinicians in predicting the future risk of CVD. In this light, we hypothesized that peripheral blood mononuclear cell (PBMC) senescence and endothelial biomarkers could influence the CV risk and could be suitable markers for the early detection of cardiovascular disease among adults with high CV risk.

## Materials & Methods

### Study Design and Setting

The study protocol was approved by the Institutional Ethics Committee (973/IEC/2016). All the study procedures were followed according to the Declaration of Helsinki. All the study participants of this cross-sectional study were screened and recruited between January 2017 and December 2017 from the General Medicine outpatient department (OPD) and hospital wards. Figure 1 provides the outline of the study.

**Figure 1:** – Flow chart representations of the research study.

### Study Subjects

This study included all adults over 18 years of age, of both genders, who received medical care at the General Medicine OPD and hospital wards with no cardiac diseases. Patients with both higher and lower cardiovascular risk were included. Patients were classified on the basis of their Interheart Risk (IHR) score. The IHR score was calculated based on the presence or absence of known CV risk factors. Patients with any cardiac disease, active immune disease, and chronic liver or kidney diseases were excluded from the study.

### Interheart Risk Score

After obtaining the informed consent form, the study participants were screened according to the inclusion/exclusion criteria, and the IHR score was measured. The IHR score was calculated using the version which did not include data on cholesterol levels. The IHR score consisted of information on medical history and data on the domains of age, gender, status with respect to diabetes, hypertension, smoking, family history of heart disease, waist-to-hip ratio, psychosocial factors, diet, and physical activity. The scores of the IHR ranged from 0 to 48, where higher and lower scores indicated higher IHR and lower IHR scores respectively. A high IHR score was defined as value16 units.^9^

### Sample Collection

Three ml of blood was obtained from the antecubital vein of the forearm in both heparin and Ethylenediaminetetraacetic acid (EDTA) vacutainers separately. The blood sample obtained in the EDTA vacutainers was subjected to centrifugation at 2,500 revolutions per minute (rpm) for 10 minutes and the isolated plasma was stored at -80°C. The blood sample collected in the heparin vacutainers were processed for the separation of Peripheral Blood Mononuclear Cell Senescence (PBMCs), using Ficoll-Histopaque reagent. The isolated PBMCs were those fixed with 70% ethanol and were stored at 4°C until further analysis.^10^Endothelial dysfunction was assessed by estimating the concentration of nitrite and of nitrate and antioxidant status.^11^

### Quantification of Total Nitrite and Nitrate

The estimation of total nitrite and nitrate was performed according to the Griess reaction assay kit using the Nitrite/Nitrate Assay Kit (Sigma-Aldrich-Catalogue Number 23479, St. Louis, USA), so as to indirectly assess the bioavailability of nitric oxide (NO). Centrifugal filters, with a molecular weight 3,000 KDa cut-off, was used to filter the plasma samples (300μl each).The analysis of the flow through plasma samples was performed using a 96-well microtiter plate, and the absorbance was read at 540nm against the reference standards.

### Estimation of Telomerase Activity

Plasma TA was estimated using the Telo TAGGG Telomerase Polymerase chain reaction (PCR) ELISA [Photometric enzyme immunoassay for the detection of telomerase activity, utilizing the Telomerase Repeat Amplification Protocol (TRAP), Roche Diagnostics GmbH, Roche Applied Science-Catalog Number 11854666910, Mannheim, Germany]. The assay was performed according to manufacturer’s instructions.

### Estimation of Total Antioxidant Capacity (TAC)

The plasma TAC was estimated using the Trolox equivalent antioxidant capacity (TEAC) assay. The analysis was performed according to manufacturer’s instructions provided in the commercially available Antioxidant Assay Kit (Sigma-Aldrich-Catalog Number CS0790, St. Louis, USA). This assay was based on the ability to determine if the presence of low molecular weight antioxidants in the plasma will inhibit the production of ABTS+ produced by the oxidation of ABTS [2, 2-Azino-bis (3-ethylbenzthiazoline-6-sulfonic acid)]. The TAC was expressed in the form of Trolox equivalents (mM).

### Fluorescence-activated Cell Sorting (FACS) Analysis

The PBMCs were isolated from the whole blood using Ficoll-Histopaque reagent. After the isolation of PBMCs, these were fixed with 70% ethanol and stored at 4°C overnight.^10^The isolated cells were then incubated for 10 minutes with RNase A (1mg/ml) for 10min at room temperature. PBMC senescence (%) was then detected using the staining regent propidium iodide by flow cytometry (FC 500 Beckmann Coulter).

### Statistical Analysis

Statistical analysis for the study was performed using SPSS 16.0 software (SPSS Inc., Chicago, IL, USA); p<0.05 was considered statistically significant. The normality of data for continuous variables was checked using Q-Q plots. Continuous variables were summarized as the mean ± standard deviation (SD), and categorical data were expressed as the frequency (Percentages). Differences in the categorical variables between groups were evaluated with the chi-square test. Parametric tests were used based on the distribution of data. Differences in continuous variables between groups were analyzed using the Independent Samples t-Test. Pearson’s correlation was performed to identify any association between the different variables. A Receiver operating curve (ROC) was plotted to identify the cut-off for all the laboratory assays so as to predict the high IHR score. A high IHR score was defined as value16 units.^9^All the necessary assumptions for performing the linear regression analysis were met. Multiple regression models were plotted to determine if the independent variable PBMC senescence, nitrite and nitrate, TAC, and TA could predict high IHR score.

## Results

### Baseline Characteristics of Study Patients

The baseline characteristics of the study patients have been illustrated ( Table 1 ). The study patients (n=60) were classified into two groups of patients with lower (n=32) and higher IHR (n=28) scores. Patients with an IHR score≥16 were classified as higher IHR score patients and those with an IHR<16 were classified as lower IHR score patients. The mean age of study patients with lower and higher IHR scores was found to be 38.09±15.82 and 43.57±11.55 years, respectively. There was no significant difference in gender among the study groups. The mean IHR scores among patients with lower and higher IHR score patients were 8.5±4.27 units and 20.46±2.19 units, respectively. As expected, the presence of CV risk factors, such as diabetes and hypertension, were greater among patients with higher IHR scores than patients with lower IHR scores.

**Table 1:** – Demographics and Risk Factors of Study Participants

Sl No.	Characteristics	Subjects with lower IHR (n=32)	Subjects with higher IHR (n=28)	p-value
1.	Age, years	38.09±15.82	43.57±11.55	0.13
2.	Male Gender, n (%)	20 (62.5%)	14 (50%)	0.33
3.	Interheart Risk (IHR) Score	8.5±4.27	20.46±2.19	0.0001
4.	Smoking, n (%)	2 (6.2%)	5 (17.9%)	0.16
5.	Diabetes, n (%)	1 (3.1)	22 (78.6%)	0.0001
6.	Hypertension, n (%)	32 (100%)	7 (25%)	0.003
7.	Family history of Heart Disease, n (%)	2 (6.2%)	4 (14.3%)	0.30
8.	Sedentary Lifestyle, n (%)	8 (25%)	9 (32.1%)	0.54

*Data was expressed as Mean ± Standard Deviation and Frequency (Percentage). The statistical tests used to compare continuous variables were the independent samples t-test, while for the categorical variables, the chi-square test was used; p-value less than 0.05 was considered statistically significant.*

### Peripheral Blood Mononuclear Cell Senescence

PBMC senescence was assessed among the study patients by estimating the TA and detecting the presence of senescent cells ( Figure 2 ). For the mean PBMC senescence (%), the percentage of senescent cells was significantly lower among patients with lower IHR scores (12.41 ± 7.40) than patients with higher IHR scores (35.26 ± 10.02) [p=0.0001] (( Figure 3a ). The mean TA (Units/3000cells) was significantly greater among patients with lower rather than higher IHR scores, [(1.80±0.53 Units/3000cells) versus (0.94±0.23 Units/3000cells) [p=0.0001] ( Figure 3b ). The presence of cardiac risk factors, such as diabetes, hypertension, and smoking, influenced the levels of PBMC senescence and TA ( Table 2 ).

**Figure 2:** – Identification and Quantification of Senescent Cells using Propidium Iodide.

**Figure 3:** – Comparision of peripheral blood mononuclear cell senescence, telomerase activity, nitrite/nitrate and total antioxidant capacity among patients with low and high interheart risk score. The statistics tests used to compare continuous variables were independent samples t-test; p-value less than 0.05 were consideres statistically significant.

**Table 2:** – Quantification of Peripheral Blood Mononuclear Cell Senescence and Endothelial Dysfunction based on the presence and absence of risk factors

	PBMC Senescence		Nitrite & Nitrate		Telomerase Activity		TAC	
Risk Factors	Presence	Absence	Presence	Absence	Presence	Absence	Presence	Absence
**Diabetes**	34.99±9.99*	15.67±11.44	204.22±42.39*	145.41±55.19	0.96±0.23*	1.67±0.60	0.52±0.08*	0.66±0.14
**Hypertension**	40.37±10.68*	20.79±13.26	224.71±28.01*	160.45±56.84	0.81±0.18*	1.47±0.59	0.40±0.09*	0.64±0.12
**Smoking**	36.05+12.38*	21.36+13.83	204.14±56.40	163.17±56.97	0.88±0.23*	1.46±0.60	0.51±0.12*	0.65±0.13
**F/H/O Heart Disease**	31.40±16.81	22.15±13.96	191.17±36.08	165.37±59.58	1.04±0.43	1.44±0.60	0.48±0.15	0.63±0.13
**Sedentary Lifestyle**	28.86±15.33	20.79±13.50	172.47±53.74	166.16±60.06	1.24±0.52	1.45±0.62	0.57±0.14	0.63±0.13

*p<0.05; PBMC: Peripheral Blood Mononuclear Cells; TAC: Total Antioxidant Capacity. Data was expressed as Mean ± Standard Deviation. The statistical tests used to compare the variables were independent samples t-tests; p-value less than 0.05 was considered statistically significant.

### Endothelial Dysfunction

The concentration of nitrite and nitrate was slightly higher among patients with higher IHR scores when compared to patients with lower IHR scores [205.14±43.60 µmole/l versus 135.41±48.95 µmole/l (p=0.0001)] ( Figure 3c ). The TAC was significantly higher among patients with lower IHR than with higher IHR scores [(0.71±0.08 mM/L) versus (0.50±0.09 mM/L) (p=0.0001] ( Figure 3d ). However, the TAC was estimated for only 30 subjects. A similar trend was observed among smokers, diabetics, and hypertensive patients ( Table 2 ).

### The Relationship Between PBMC Senescence and Endothelial Dysfunction

We observed a significant positive correlation between age and PBMC senescence (r=0.36, p=0.005), but a significant negative correlation was observed between age and TAC (r=-0.60, p=0.0001). IHR scores demonstrated significant positive correlations with PBMC senescence (r=0.75, p=0.0001) and nitrite & nitrate (r=0.56, p=0.0001), whereas significant negative correlations were observed with TA (r=-0.83, p=0.0001) and TAC (r=-0.92, p=0.0001). Additionally, PBMC senescence also showed significant correlations with the variables nitrite and nitrate, TAC, and telomerase activity ( Figure 4 ).

**Figure 4:** – Correlation of PBMC senescence with age, nitrite/nitrate, telomerase activity and total antioxidant capacity.

ROC Curve Analysis for PBMC Senescence and Endothelial Dysfunction:

The ROC curve was plotted to check if PBMC senescence, nitrite and nitrate, antioxidant status, and TA could predict high IHR scores among the studied patients. The analysis demonstrated that PBMC senescence of 0.95 [p-value = 0.0001; 95% CI (0.874-1.026)] was a significant predictor of patients with higher IHR scores, with a cut-off value of 21.65, and with a sensitivity and specificity of 92% and 88%, respectively ( Figure 5 ).

**Figure 5:** – Receiver operating characteristic curves for the prediction of High Interheart Risk Score.

### Multiple Regression Models for PBMC Senescence and Endothelial dysfunction

Multiple regression models were plotted to analyze the effect of the independent variables of PBMC senescence, nitrite and nitrate, and TA on the dependent variable IHR score ( Table 3 ). It was observed that PBMC senescence, nitrate and nitrite, and TA were independently associated with high IHR scores ( Table 3 ).

**Table 3:** – Multiple Regression Models to predict Interheart Risk Score

		Coefficients						
Model			Unstandardized Coefficients		Standardized Coefficients	t	Adjusted R Square	p-value
			B	Std. Error	Beta			
1	(Constant)		27.449	1.295		21.197	0.679	0.0001
	TelomeraseActivity		-9.575	.854	-0.827	-11.216		.000
2	(Constant)		21.160	2.470		8.567	.716	0.0001
	TelomeraseActivity		-8.357	.904	-0.722	-9.242		0.0001
	NitriteandNitrate		.027	.009	0.229	2.927		0.005
3	(Constant)		17.112	2.988		5.727	.735	0.000
	TelomeraseActivity		-6.608	1.169	-0.571	-5.652		0.000
	Nitrite and Nitrate		.021	.009	0.179	2.274		0.027
	Senescence		.113	.050	0.235	2.251		0.028

B: Unstandardized Regression Coefficient; SEβ: Standard Error of Coefficient; β: Standardized Coefficient; p<0.05*

## Discussion

The relationship between PBMC senescence and endothelial dysfunction, and the occurrence of CVD has been described in previous studies;^3 , 12^however, the information regarding the relationship between PBMC senescence, endothelial dysfunction, and CVD among subjects with no established CVD remains sparse. To the best of our understanding, this is the first clinical study conducted in the South Indian population that estimated PBMC senescence and determined its relationship with high CV risk using the IHR score. The main finding of our study was that PBMC senescence, nitrite and nitrate, and TA were independently associated with high IHR scores. The severity of PBMC senescence was greater among patients with higher CV risk when compared to patients with lower CV risk. PBMC senescence was estimated on the basis of TA and the percentage of senescent cells (%) among the studied patients.

Telomeres and telomerase play a significant role in the development and pathogenesis of CVD. It is well-known that, with each cell division, the length of telomeres shortens, whereas inflammation and oxidative stress, which are major mechanisms involved in the development and pathogenesis of CVD, are known to increase the rate of telomere shortening, leading to cell senescence.^13 - 15^Moreover, the presence of lower TA and shorter leukocyte telomere length (LTL) has been seen in the senescent endothelial cells, vascular smooth muscle cells (VSMCs), and atherosclerotic plaque, and these are also associated with plaque instability leading to CVD. The absence of TA, which maintains the telomere integrity and telomere length, makes the cell senescent and causes apoptosis.^16 - 18^Our study revealed that TA was significantly lower among patients with higher than lower IHR scores. In contrast to our findings, an earlier study, named coronary artery risk development in young adults (CARDIA), conducted among young patients with coronary artery risk development with prevalent coronary artery calcium (CAC), revealed that TA plays a vital role in the development of atherosclerosis. The findings of the study demonstrated that higher levels of telomerase predicted a higher prevalence of CAC among young to middle-aged men. However, patients with shorter telomere length presented a positive association between TA and CAC.^19^In an earlier cross-sectional study, the association between subclinical atherosclerosis burden and both average LTL and the abundance of short telomeres (%LTL<3 kb) was studied among 4,066 asymptomatic middle-aged subjects without the presence of any CVD. The study showed that the average LTL and short telomeres were not significant and independent predictors of subclinical atherosclerosis.^20^In one of the largest observational and genetic studies, conducted in 290,022 individuals from Copenhagen, it was revealed that the presence of short telomeres was associated with a higher risk of ischemic heart disease.^21^The differences in the study findings might be attributable to the heterogeneity observed in the study population and the sample size of the study. Moreover, a recent systematic review and meta-analysis of twenty-four studies revealed an inverse association between leukocyte telomere length and the risk of coronary heart disease (CHD), regardless of conventional vascular risk factors.^3^The systematic review included cardiovascular patients, whereas our study included patients free of CVD. Therefore, it can be suggested that measuring TA and LTL might be a useful marker for predicting the future risk of CVD. Presently, investigations are being carried out to gauge if statins could be used as potential therapeutic agents for telomerase activation and as effective geroprotectors.^22^

Lately, senescent cells have gained attention as a therapeutic target for several age-related diseases, such as CVD. Studies have shown that cell senescence has been equivalent to endothelial senescence, and thus vascular senescence as well. The present study then measured the percentage of senescent cells (%), which was significantly lower among patients with lower IHR scores, when compared to those with higher IHR scores. The transcriptional analysis of human VSMCs demonstrated that there was a suppression of the matrix Gla protein (MGP), an inhibitor of calcification, in the senescent VSMCs. Furthermore, there was also an up-regulation of transcript encoding bone morphogenic protein 2 (BMP2), which is a promoter of calcification.^23^Therefore, it can be suggested that the senescent VSMCs might play a prominent role in the development of age-related hardening and stiffening through increased calcification. The stiffening and hardening of arteries lead to the development of high blood pressure, which is considered to be one of the major risk factors for the occurrence of coronary artery disease, HF, stroke, and MI.^24^Another study conducted to compare MGP expression in normal versus diseased aortic valve interstitial cells (AVICs) showed that the MGP expression was significantly decreased in the diseased AVICs relative to normal AVICs. These findings imply that the absence of an anti-calcification defense mechanism might contribute to the calcification of the aortic valve.^25^Therefore, estimating the percentage of senescent cells might be a potential and novel marker for predicting the development and progression of CVD. Novel therapeutic strategies that involve the prevention, removal, and replacement of the senescent cells are at their inception. Further understanding and more research are required to understand this biology so as to translate this knowledge into therapeutic applications.

The present study measured endothelial dysfunction by estimating the concentration of nitrite and nitrate and the TAC. TAC was found to be significantly lower among patients with higher IHR scores when compared to patients with lower IHR scores. Several epidemiological studies have demonstrated that people with a higher intake of antioxidant vitamins have a lower risk of developing MI and stroke.^26 , 27^However, a recent systematic review and meta-analysis of randomized controlled trials revealed that the current literature provided no evidence to support the use of vitamins and antioxidants for the prevention of CVD.^28^However, a recent systematic review of observational studies demonstrated a substantial association between higher levels of dietary total antioxidant capacity and risk factors of cardiovascular diseases.^29^Our study also showed that the nitrite and nitrate concentrations were higher among high-risk patients when compared to low-risk patients. In contrast, the Framingham offspring study demonstrated that a higher plasma nitrate concentration was associated with all-cause mortality but was not found to be associated with the incidence of CVD.^30^This might be due to the fact that the nitrite and nitrate concentrations present in the diet could be metabolized into NO, thereby promoting cytoprotection and cardiovascular benefits.^31^The results in our study might be contrasting due to the fact that certain diets, such as vegetables, fruits, and processed meats, are rich sources of nitrites and nitrates.^32^Hence, there are possibilities that high risk patients in our study had been exposed to such diets. The endothelial-dependent response to vasodilation is regulated by the release of NO synthesized from the dietary nitrate, nitrite and amino acid L-arginine, via the endothelial nitric oxide synthase (eNOS) ,which leads to the production of intracellular cyclic GMP. However, endothelial dysfunction leads to the imbalance in the production of NO and ROS, in turn leading to the occurrence of several age-related diseases, such as CVD. The accumulation of ROS in the arterial plasma and intima leads to an increase in the low-density lipoprotein (LDL) oxidation; the uptake of this oxidized LDL by the arterial macrophages is one of the prominent factors for the formation and progression of atherosclerotic plaque. Therefore, the presence of antioxidants in the plasma, LDL particle, and cell wall can inhibit the LDL oxidation and can safeguard the vasoreactivity by increasing the release of endothelial NO and by reducing thrombogenicity.^12 ,33^Therefore, determining the TAC and the concentration of nitrite and nitrate can turn out to be a potential marker for the early prediction of CVD in the future.

### Limitations

The main limitation of our study is with respect to the limited sample size. Another limitation is that our study did not have a prospective long-term follow-up with the confirmation of clinical events; instead, we calculated the risk based on the interheart risk score. Additionally, the blood samples were collected at different time points, which could have had an effect on the levels of laboratory assays.

## Conclusions

Our study demonstrated that PBMC senescence, TA, and nitrite and nitrate are suitable measures to predict high cardiovascular risk in adults with CV risk. Therefore, measurements of the above markers might be used as an additional risk assessment tool to predict the risk of cardiovascular diseases among adults. Nevertheless, long-term prospective follow-up studies with the adjudication of clinical events are required to confirm these findings.
